# Should screening for *Chlamydia trachomatis* and *Neisseria gonorrhoeae* in HIV-men who have sex with men be recommended?

**DOI:** 10.7448/IAS.17.4.19661

**Published:** 2014-11-02

**Authors:** Isabel Pérez-Hernández, Rosario Palacios, Carmen González-Doménech, Victoria García, Manuel Márquez, Encarnación Clavijo, Jesús Santos

**Affiliations:** 1 Infectious Diseases Unit, Hospital Virgen de la Victoria, Málaga, Spain; 2 Microbiology, Hospital Virgen de la Victoria, Málaga, Spain

## Abstract

**Introduction:**

Sexually transmitted infections (STI) like *Chlamydia trachomatis* (CT) and *Neisseria gonorrhoeae* (NG) have been associated with increased risk of HIV acquisition [1]. It has been also described as a high prevalence of asymptomatic CT and NG infections in men who have sex with men (MSM) [2]. The aim of this study was to know the prevalence of CT and/or NG infections in asymptomatic HIV-MSM and the related factors.

**Materials and Methods:**

Prospective study of a cohort of asymptomatic HIV-MSM with follow-up in Malaga (southern Spain) during October 2012–May 2014. Patients with an opportunistic event or who received active antibiotic therapy for CT and/or NG in the previous month were excluded. All of them completed a questionnaire about sexual behaviour, barrier methods and recreational drugs use. Demographical, epidemiological, clinical, analytical and therapeutic data were also collected. Pharyngeal and rectal swabs, and urine samples were collected to be tested for CT and NG by nucleic acid amplification test (c4800 CT/NG. Roche Diagnostics, Mannheim, Germany) [3]. Statistics analysis: SPSS 17.0.

**Results:**

255 patients were asked to participate and 248 of them accepted. Median age was 37.7 (30.6–46.3) years, median time since HIV diagnosis was 47.7 (10.5–104.1) months, and median CD4 cells count was 607 (440–824) cell/µL. There were 195 (78.6%) patients on antiretroviral therapy; 81.5% of them had undetectable viral load. 80.5% of the patients had a past history of STI. Infection by CT and/or NG was diagnosed in 24 (9.7%) patients. Overall four urine samples, two pharyngeal, and 15 rectal ones were positive for CT, and five pharyngeal and five rectal swabs were positive for NG. Two patients were co-infected by CT and NG: one with CT in urine and both in rectum, another with CT in urine and rectum and NG in pharynx. One patient presented CT in pharynx and rectum, and two patients NG in pharynx and rectum. Positive CT and/or NG tests were only related with detectable HIV viral load (OR 3.08, 95% CI 1.2–7.4; p=0.01). It was not related with sexual behaviour, nor with alcohol or recreational drugs use.

**Conclusions:**

STI screening had a great acceptance in this population. There was a high prevalence of asymptomatic CT and/or NG infections. Rectum sample was the most effective one. Viral suppression could protect from these STI. Screening should be recommended in HIV-MSM.

## References

[1] Bernstein K, Marcus J, Nieri G, Philip SS, Klausner JD (2010). Rectal gonorrhea and chlamydia reinfection is associated with increased risk of HIV seroconversion. AIDS.

[2] Benn PD, Rooney G, Carder C, Brown M, Stevenson SR, Copas A (2007). *Chlamydia trachomatis* and *Neisseria gonorrhoeae* infection and the sexual behaviour of men who have sex with men. Sex Transm Infect.

[3] Rockett R, Goire N, Limnios A, Turra M, Higgens G, Lambert SB (2010). Evaluation of the cobas 4800 CT/NG test for detecting *Chlamydia trachomatis* and *Neisseria gonorrhoeae*. Sex Transm Infect.

